# Theoretical Studies of a Silica Functionalized Acrylamide for Calcium Scale Inhibition

**DOI:** 10.3390/polym14122333

**Published:** 2022-06-09

**Authors:** Abdulmujeeb T. Onawole, Ibnelwaleed A. Hussein, Mohammed A. Saad, Nadhem Ismail, Ali Alshami, Mustafa S. Nasser

**Affiliations:** 1 Gas Processing Center, College of Engineering, Qatar University, Doha P.O. Box 2713, Qatar; a.onawole@qu.edu.qa (A.T.O.); m.saleh@qu.edu.qa (M.A.S.); m.nasser@qu.edu.qa (M.S.N.); 2 Chemical Engineering Department, College of Engineering, Qatar University, Doha P.O. Box 2713, Qatar; 3 Department of Chemical Engineering, University of North Dakota, Grand Forks, ND 58202, USA; nadhem.ismail@und.edu

**Keywords:** acrylamide functionalized silica, DFT study, Ab Initio Molecular Dynamics, oilfield scales

## Abstract

The calcium carbonate (CaCO_3_) scale is one of the most common oilfield scales and oil and gas production bane. CaCO_3_ scale can lead to a sudden halt in production or, worst-case scenario, accidents; therefore, CaCO_3_ scale formation prevention is essential for the oil and gas industry. Scale inhibitors are chemicals that can mitigate this problem. We used two popular theoretical techniques in this study: Density Functional Theory (DFT) and Ab Initio Molecular Dynamics (AIMD). The objective was to investigate the inhibitory abilities of mixed oligomers, specifically acrylamide functionalized silica (AM-Silica). DFT studies indicate that Ca^2+^ does not bind readily to acryl acid and acrylamide; however, it has a good binding affinity with PAM and Silica functionalized PAM. The highest binding affinity occurs in the silica region and not the –CONH functional groups. AIMD calculations corroborate the DFT studies, as observed from the MD trajectory that Ca^2+^ binds to PAM-Silica by forming bonds with silicon; however, Ca^2+^ initially forms a bond with silicon in the presence of water molecules. This bonding does not last long, and it subsequently bonds with the oxygen atoms present in the water molecule. PAM-Silica is a suitable calcium scale inhibitor because of its high binding affinity with Ca^2+^. Theoretical studies (DFT and AIMD) have provided atomic insights on how AM-Silica could be used as an efficient scale inhibitor.

## 1. Introduction

Scale deposition is the bane of the oil and gas industry. These oilfield scales often contain calcium carbonates and sulfates, including barium sulfate, iron sulfides, and many others [1,2]. Calcium carbonate (CaCO_3_) is one of the most frequently occurring scales [3]. This scaling can occur during oil production, stimulation, and even transportation. CaCO_3_ scale formation occurs when ${\mathrm{Ca}}^{2+}$ is supersaturated with ${\mathrm{CO}}_{3}{}^{2-}$ or ${\mathrm{HCO}}_{3}{}^{-}$ carbonates, as illustrated in Equations (1) and (2):

(1) $${\mathrm{Ca}}^{2+}{}_{\left(\mathrm{aq}\right)}+{\;\mathrm{CO}}_{3}{{}^{2-}}_{\left(\mathrm{aq}\right)}\to {\mathrm{CaCO}}_{3}{}_{\left(s\right)}$$

(2) $${\mathrm{Ca}}^{2+}{}_{\left(\mathrm{aq}\right)}+2{\mathrm{HCO}}_{3}{{}^{-}}_{\left(\mathrm{aq}\right)}\to {\mathrm{CaCO}}_{3}{}_{\left(s\right)}+{\mathrm{CO}}_{2}{}_{\left(g\right)}+H_{2}O_{\left(l\right)}$$

Different factors cause ion supersaturation. Seawater can react with the formation water in offshore fields such as the North Sea, leading to CaCO_3_ deposits that hinder oil production, which is a significant problem [4]. The High Pressure and High Temperature (HPHT) operating conditions during the production process also contribute to scaling deposition [5] 

The consequences of calcium scale deposition can bring production capacity to a halt in a few hours, with a very high treatment cost [6]. A notable example is the North Sea Miller oilfield incident, where scale deposition decreased production from 30,000 barrels of oil per day to zero in just one day [7,8]. Other issues caused by scale include pipe blockage, which hinders fluid flow; damage to subsurface equipment; and increased corrosion rates [9,10]. These problems can lead to severe accidents that affect production safety and economics [11]. 

Calcium scales can be removed by mechanical or chemical means. Mechanical removal refers to physical deposit removal. This method can exacerbate the situation by aiding the rate of corrosion, often known as pitting corrosion since tiny holes are made in the metal pipes during cleaning [1]. Some scales, such as calcium sulfate, are impossible to remove mechanically [5]. Chemical removal is low in cost and can be used to remove scales that are not physically accessible; however, some chemicals, such as hydrochloric acid, can worsen the situation by producing corrosive hydrogen sulfide gas. A better approach for scale mitigation would be prevention or scale inhibition. This approach ensures continuous production, unlike removal, which requires a stop in production. 

Many types of scale inhibitors (SI) have been used in the last decade to hinder CaCO_3_ scale [12,13,14,15]. Scale inhibitors often function in two ways: an adsorption effect or a morphological change in the growing site. Adsorption effects occur when the inhibitor occupies the nucleation sites; therefore, the crystals cannot find surfaces to adhere to and will not grow. The morphological change method occurs when the inhibitor is adsorbed, which changes the scale’s morphology, making it difficult for the scale to remain and grow [4]. SIs are often categorized as organic or inorganic inhibitors. Phosphates and their salts are often inorganic. Polymers, such as polyacrylic acid (PAA) and sulfonated polymers, and copolymers containing sulfonated and phosphonated moieties are examples of organic SIs [16]. The primary aim of the chemical inhibitors is to sequester the calcium ion; therefore, they prevent it from reacting with carbonate or bicarbonate ions, which prevents calcium carbonate scale formation [16,17,18]. 

Polyacrylamide (PAM) is used extensively in the oil industry for enhanced oil recovery. This substance is used as a flocculant in water treatments due to its ability to bridge particles and form aggregates with good settling properties [19,20]. PAM’s amount of branching and charge density and its flocculation and adsorption capacity increase when the molecular weight is increased, enhancing its overall performance [21]. PAM hydrolysis results in the generation of more carboxylic groups at high temperatures, which act as Ca^2+^ adsorption or binding sites.

Polymer functionalization improves material performance [22]. Silica (SiO_2_) has been mixed with PAM to create adsorbents, partly due to its large surface area coupled with its stability and ease of modification. Functionalization has been applied to removing heavy metals such as mercury (Hg) and lead (Pb) [23,24]. Silicon’s negative charge may act as a nucleation site for the metal ions. 

Theoretical studies have provided exceptional insights on the atomistic scale that aids in understanding chemical process mechanisms. This application has also gained wide use in oilfield chemistry, particularly in scale removal [9,25,26,27,28]. Density Functional Theory (DFT) and Ab Initio Molecular Dynamics (AIMD) are reliable theoretical techniques that provide accurate results for understanding chemical systems. 

DFT and AIMD were used in this study to understand the Ca^2+^ removal process using mixed oligomers of acrylic acid, acrylamide, and PAM functionalized silica (PAM-Silica). Due to the computational costs of the methods employed. The PAM model used was simplified based on its functional property (-CH_2_CHCONH_2_-). These insights will allow us to design better polymer-based scale inhibitors, assisting in calcium-based scale formation prevention, previously studied experimentally by our group [29]. The binding affinities of these polymers to Ca^2+^ were studied alongside other quantum chemical parameters, such as molecular electrostatic potential (MEP) and frontier molecular orbital (FMO) maps using DFT. Also, QTAIM analysis was done to validate the binding energies observed in the DFT calculations. AIMD calculations provide detailed information about the movement of the atoms and molecules in the chemical system, with the most promising results from the DFT calculations to better understand how they interact. Combining these two techniques gives an accurate and detailed understanding of Ca^2+^ adsorption by the studied polymers, including silica functionalized PAM. 

## 2. Computational Details

### 2.1. Quantum Chemical Calculations

The Gaussian 09 code [30] was used for all DFT quantum calculations. All calculations were completed using the B3LYP level of theory with the TZVP basis set. The level of theory B3LYP is robust and well-known in theoretical scale inhibitor studies since it gives reliable results with reasonable computational costs [31,32]. We chose TZVP (triple-zeta valence with polarization) to minimize basis set superposition errors, despite it being computationally expensive compared to other reliable basis sets such as 631g-(d) [33,34]. All calculations were performed using water as a solvent with the PCM-SCRF (Polarizable Continuum Model-Self-Consistent Reaction Field). Vibrational frequencies were calculated, and the absence of negative values implied true minima and no imaginary frequency present. Gauss View 5.0 [35,36] was used to visualize molecular electrostatic and frontier molecular orbital maps. The Quantum Theory Atom In Molecules (QTAIM) developed by Bader [37] was done on the optimized structures from Gaussian 09 to validate the binding interactions with the aid of Multiwfn program [38]. QTAIM analysis has proven to be a useful tool in analyzing bond interactions [39]. The VMD program [40] was employed for the visualization of the QTAIM Bond Critical Points (BCP). 

### 2.2. AIMD Calculations

The Ab Initio Molecular Dynamics (AIMD) calculations were performed with VASP code (v5.4.4) [41,42,43] using periodic boundary conditions (PBC). The Projected-Augmented-Waves Perdew-Burke-Ernzerhof (PAW-PBE) pseudopotentials [44] under the Generalized Gradient Approximation (GGA) exchange form of correlation were used for all elements in the system studied. The temperature was set to begin at 298 K and increased to 373 K at a 1.25 × 10^−4^ K/ps rate since many operating conditions in the oil industry are approximately 373 K. The number of ionic steps and time steps were 20,000 and 0.5 fs, respectively, with a total of 10 ps simulation time. However, in following best practices, the first 2 ps was not considered in the trajectory to enable the system get equilibrated. The inclusion of Grimme’s DFT+D3 [45,46,47] was included in the calculation has it has shown to improve the accuracy of the results by describing the dispersion forces in the studied system. 

The implicit solvent was considered by implementing the VASPsol models [27,48]. This was done to simulate a realistic model. This method has proved to be reliable as it is better than the Finite-Difference Poisson Boltzmann (FDPB) approach and on par with the Solvation Model Density (SMD) approach [49]. Moreover, this method has also been applied to polymer systems [50] Though it is important to note that the perfect simulation of reality based on quantum-mechanical description remains a tricky situation [51]. A 1 × 1 × 1 Gamma (γ) grid was employed for the *k*-point and the planewave energy cut-off was set to the default which is the largest ENMAX in the POTCAR. The Quantum ATK Virtual NanoLab builder and MD analyzer [52] were used to build and visualize the model. 

## 3. Results and Discussion

### 3.1. Binding Affinities

**Figure 1:**
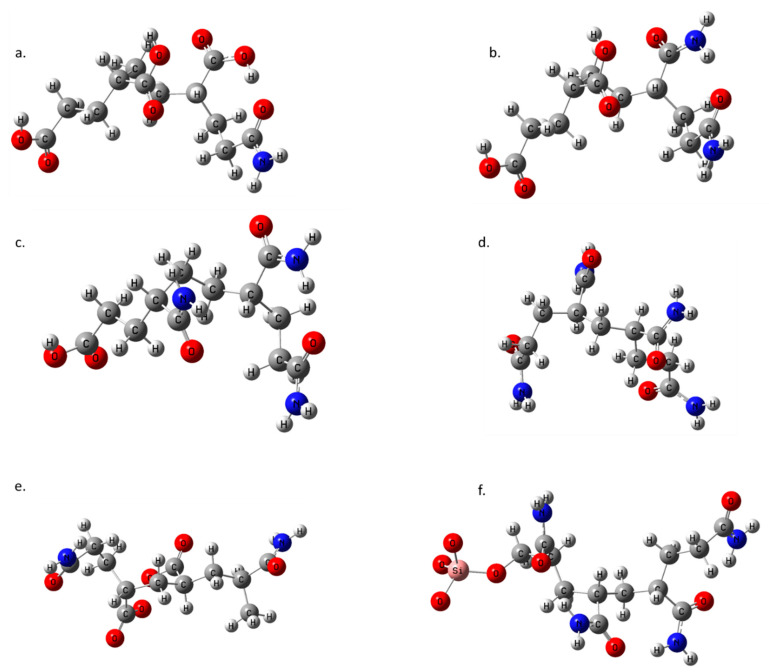
The optimized structures of (**a**) AA-AA-AA-AM (70%-Acrylic Acid), (**b**) AA-AA-AM-AM (50% each of acryl acid and acryl amide), (**c**) AA-AM-AM-AM (70% acryl amide), (**d**) PAM, (**e**) PAM_hydrolyzed, and (**f**) PAM-Silica.

The Ca^2+^ ion was complexed with the DFT optimized structures of six oligomers (Figure 1). The binding affinities of these oligomers were calculated using Equation (3). The first three sets of oligomers were a mix of acryl acid (AA) and acrylamide (AM) monomers. The first oligomer contained 70% acryl acid. Three of the four monomer units were acryl acid, and the last was acrylamide (AA-AA-AA-AM). The second oligomer had 50% each acryl acid and acrylamide (AA-AA-AM-AM), while the third oligomer contained three units of acrylamide and one unit of acryl acid (AA-AM-AM-AM). The mixed oligomers all had positive values, denoting that the binding affinity with the Ca^2+^ ion was weak.

(3) $$\Delta E_{binding\;energy}=\Delta E_{Ca-complex}-(\Delta E_{Ca^{2+}}+\Delta E_{complex})$$

**Figure 2:**
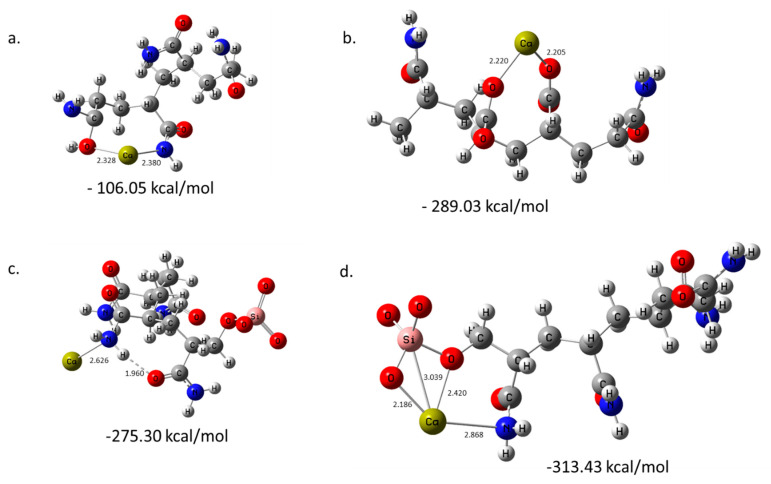
The optimized structures and binding energies of (**a**) Ca-PAM, (**b**) Ca-PAM_ hydrolyzed, (**c**) Ca-PAM_Silica1, and (**d**) Ca-PAM_Silica2. All bond lengths are in Angstroms.

The remaining three oligomers (Figure 2) had negative values, denoting good binding affinity with the Ca^2+^ ion. The fourth oligomer comprised four acrylamide (PAM) units and had a binding affinity of −106.05 kcal/mol (Figure 2a). AM oligomer formed two bonds with Ca^2+^: the Ca-O bond with a bond length of 2.328 Å, and Ca-N with a length of 2.380 Å. The fifth oligomer of the hydrolyzed_PAM had a binding affinity of −289.03 kcal/mol, which is more than double the binding affinity for PAM alone; however, this double-fold increase is possibly due to the bond type formed. Two bonds are formed in hydrolyzed_PAM; however, the two bonds formed are Ca-O bonds with bond lengths of 2.220 Å and 2.205 Å, unlike PAM, where one of the bonds formed was Ca-N. These results imply that Ca-O bonds are shorter and stronger than Ca-N bonds. A silica-functionalized AM was the last oligomer studied; however, the binding affinities were studied at two positions. The first position studied was the binding affinity of PAM_Silica with Ca^2+^ ions using the oxygen and nitrogen atoms (Figure 2c). The binding affinity was −275.30 kcal/mol. This complex only formed one bond: Ca-N (2.626 Å).

Nevertheless, its relatively high binding affinity compared to ordinary PAM (−106.05 kcal/mol) is possible due to intra-hydrogen bonding, which contributes positively to its binding affinity. The second position studied the binding of Ca^2+^ to the silica part of the oligomer (Figure 2d). The binding affinity calculated was the highest amongst all studied oligomers (−313.43 kcal/mol). Ca^2+^ was within the bonding distance of four atoms, including two Ca-O bonds with 2.185 Å and 2.420 Å bond lengths, one Ca-N bond (2.868 Å), and a Ca-Si bond (3.039 Å). The presence of these bonds is responsible for its high binding affinity, implying that functionalizing PAM with silica will significantly increase its binding affinity to Ca^2+^ ions. These results agree with what has been observed, where the adsorption of SiO_2_-PAM on Ca^2+^ is a spontaneous process, primarily due to chemical interaction and chelation [53].

(4) $$\Delta E_{binding\;energy}=\Delta E_{Ca-complex}-(\Delta E_{Ca^{2+}}+\Delta E_{complex})$$

### 3.2. QTAIM Analysis

**Figure 3:**
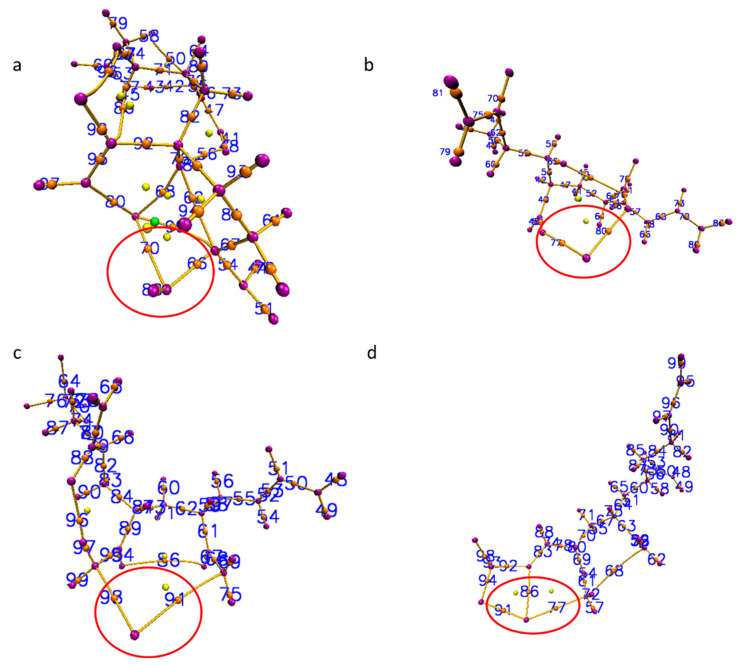
The bond critical points (BCP) of (**a**) Ca-PAM, (**b**) Ca-PAM_ hydrolyzed, (**c**) Ca-PAM_Silica1, and (**d**) Ca-PAM_Silica2. The BCP in red circles are those of concern that form bonds with Ca^2+^.

The QTAIM analysis confirmed the presence of 3, 2, 2, 3 BCPs (3, −1) for Ca-PAM, Ca-PAM-hydro, Ca-PAM-Silica I and Ca-PAM Silica_2 respectively (Table 1). The DFT calculated energy values and binding energies for all the studied complexes can be seen in the supplemental information’s Table S1. The BCPs are illustrated in red circles in Figure 3. Usually, low electron density (ρ(r)) −0.001 to −0.059 a.u, positive values of the Laplacian (∇^2^ ρ(r)), and zero or near zero energy density (H_b_)- 0.000 to 0.005 a.u. at BCPs denote typical non-covalent interactions [54,55]. The interaction energies which are defined based on the works of Espinosa et al. [56] and Vener et al. [57] for E_int_^a^ and E_int_^b^ respectively show that non-covalent interactions exist between Ca-H in Ca-PAM and Ca-N in Ca-PAM_Slica1 and Ca-PAM_Silica 2. However, the high interaction energy observed in Ca-PAM_Silica 2 implies a degree of covalency and this corroborates the high binding energy observed from the DFT calculations. The trend in both the QTAIM interactions enegies correlates with the same trend observed in the binding energy calculation, with Ca-PAM_Silica2 having the highest in both cases. There is no significant difference between the E_int_^a^ and E_int_^b^ except in Ca-PAM_Silica 2, where E_int_^a^ is about 3 kcal/mol higher than E_int_^b^.

### 3.3. Quantum Chemical Analysis

**Figure 4:**
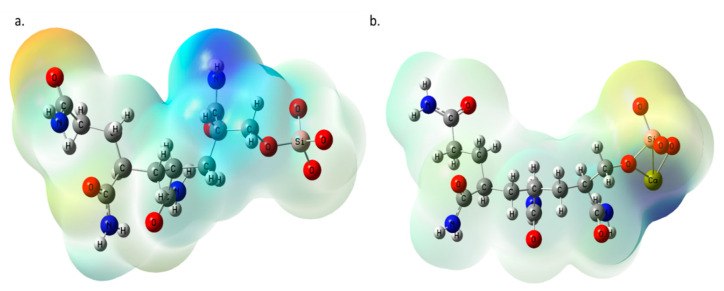
The molecular electrostatic potential maps for (**a**) PAM-Silica and (**b**) Ca-PAM_Silica2.

The electronic structure calculations from DFT give rise to other interesting studies, which provide atomistic insights into how molecules are formed. These insights include the molecular electrostatic potential (MEP) and frontier molecular orbitals (FMO) maps. The MEP denotes the charge distribution within the molecule, designated by yellow, orange, and red, with red being the most electronegative. Regions of electropositivity are represented as green, blue, and violet, with violet corresponding to the most electropositive region. The MEP maps of the silica functionalized PAM before and after binding with Ca^2+^ are compared in Figure 4. The MEP before the binding is primarily electronegative, with the oxygen atoms appearing in the yellow region except for where silicon is located, which seems very light yellow. This change in electronegativity is possibly a neutralization effect by the silicon atom since it is less electronegative than the surrounding oxygen atoms, which are highly electronegative. 

A small blue region denotes electropositivity on the hydrogen atom of the –CONH functional group preceding silica. This electropositivity is possibly due to the close proximity of the silicon atom to the –CONH functional group. This group is also coupled with the many carbon and hydrogen atoms that are less electronegative; therefore, the high electronegativity of the nitrogen and oxygen atoms of the –CONH group is neutralized. The second position has the highest binding affinity, involving a Ca-Si bond. The MEP map for Ca-PAM-Silica indicates a solid electropositive (deep blue) region at the location of Ca^2+^ and its surrounding atoms (silicon and oxygen), indicating that Ca^2+^ is electropositive and forms strong bonds with the surrounding oxygen atoms, dominating the oxygen atoms’ electronegativity. A yellow region is located where the only oxygen atom (SiO_4_^−^) is not binding to Ca^2+^, indicating that this oxygen atom still retains its high electronegativity. The electronegativity is somewhat neutralized; therefore, the color is yellow instead of red due to its nearness to Ca^2+^. The rest of the molecule is primarily light blue, denoting an overall electropositive charge. 

**Figure 5:**
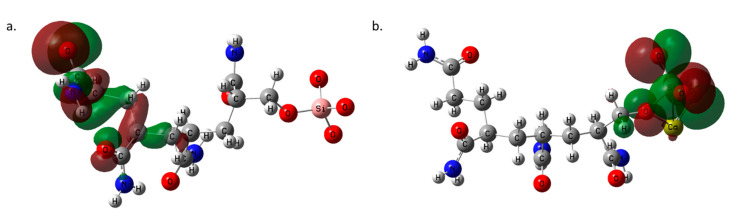
The Highest Occupied Molecular Orbital (HOMO) maps for (**a**) PAM-Silica and (**b**) Ca-PAM_Silica.

**Figure 6:**
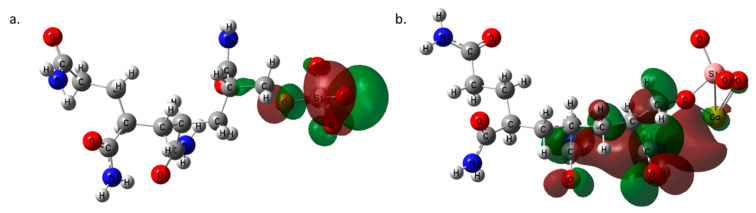
The Lowest Unoccupied Molecular Orbital (LUMO) maps for (**a**) PAM-Silica and (**b**) Ca-PAM_Silica.

The Highest Occupied Molecular Orbital (HOMO) and Lowest Unoccupied Molecular Orbital (LUMO) make up the Frontier Molecular (FMO) Orbitals. These orbitals give insight into the electron delocalization of the electrons in the molecule. The PAM HOMO map indicates that the electrons are delocalized at the –CONH end of the molecule (Figure 5a). Electron delocalization upon binding with Ca^2+^ occurs where Ca^2+^ bonds with the silicon and oxygen atoms, denoting electron exchange (Figure 5b). The LUMO of PAM-Silica (Figure 6a) reveals that the electrons are mostly delocalized at the silica region, similar to the HOMO of PAM-Silica when bound to Ca^2+^ (Figure 5b). This phenomenon indicates that the HOMO and LUMO of PAM-Silica occur at both molecule ends. The electrons are delocalized at the atoms close to the calcium bonded region when the LUMO of PAM-Silica is bound to Ca^2+^ (Figure 6b). The number of lobes is higher than ordinary PAM-silica alone in the HOMO and LUMO maps of the Ca^2+^ bonded PAM-Silica, implying that electrons are more delocalized when PAM-Silica bounds to Ca^2+^.

### 3.4. AIMD Analysis

**Figure 7:**
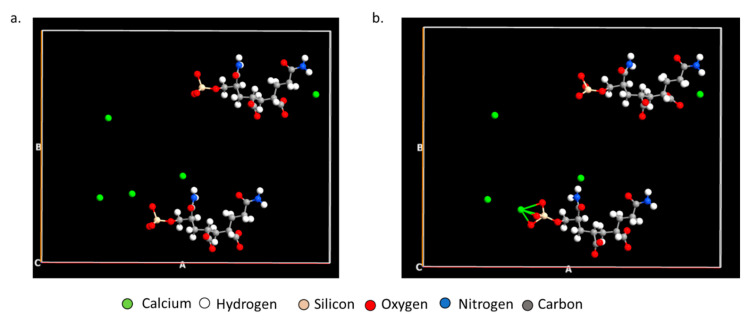
The (**a**) initial and (**b**) final position of the MD trajectory of PAM-Silica with Ca^2+^ ions.

**Figure 8:**
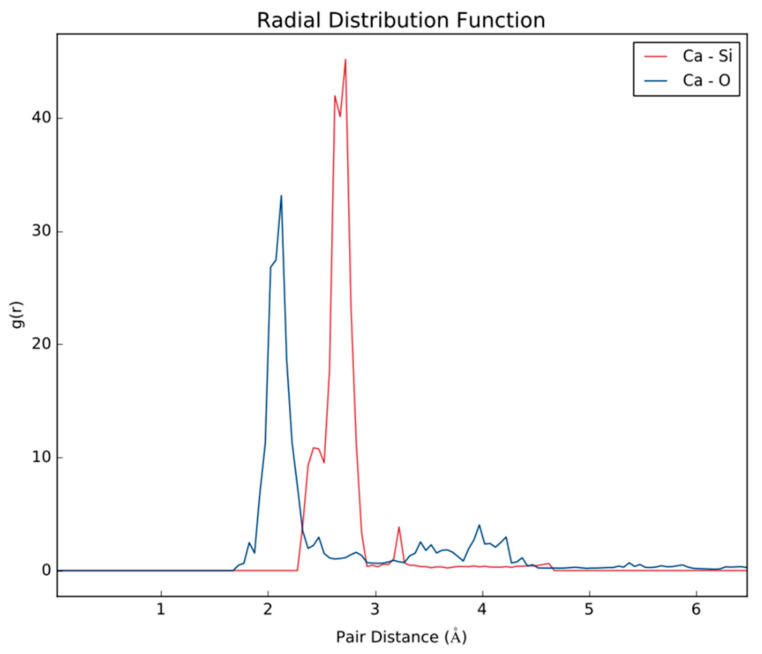
The radial distribution function of the MD trajectory of PAM-Silica with Ca^2+^ ions.

*Ab Initio* Molecular Dynamics (AIMD) further establishes how PAM-Silica would interact with calcium ions by observing the time evolution in the trajectory. Two AIMD systems were studied: the first system studied the interaction between PAM-Silica and Ca^2+^ ions. In contrast, the second system was similar to the first system but included explicit water molecules. The MD trajectory analysis confirmed earlier observations from the DFT studies that Ca^2+^ would preferentially bind to the silica region instead of the –CONH functional groups (Figure 7b). Ca forms Ca-O and Ca-Si bonds, as observed earlier from DFT. The radial distribution function (RDF), which is the probability of finding an atom at a distance *r* from another tagged atom, was analyzed between Ca-O and Ca-Si atoms (Figure 8). The RDF indicated that both Ca-Si and Ca-O were within bonding distance, less than 3 Å; however, Ca-Si has a more prominent and higher peak, implying that the probability of occurrence for the Ca-Si bond is higher than the Ca-O bonds. 

**Figure 9:**
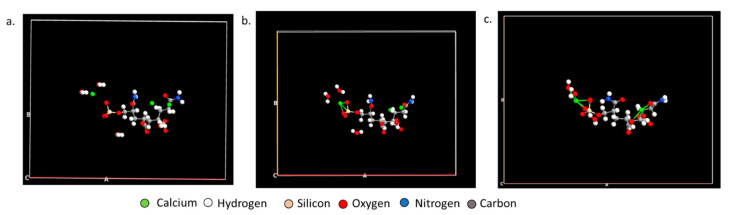
The (**a**) initial, (**b**) middle, and (**c**) final position of the molecular dynamics trajectory of PAM-Silica with Ca^2+^ ions and water molecules.

**Figure 10:**
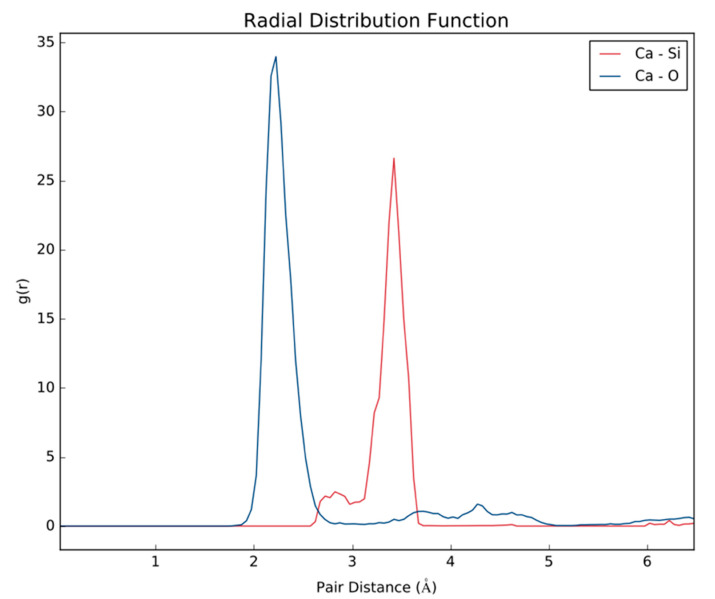
The radial distribution function (RDF) of the MD trajectory of PAM-Silica with Ca^2+^ ions and water molecules.

Table 2 ions were put in closer proximity to PAM-Silica to enable easier binding since AIMD is computationally expensive and can only be observed for a few picoseconds. The initial stage depicts two water molecules and one Ca^2+^ near PAM-Silica (Figure 8a); however, Ca^2+^ first bonds to the silica region, not the water molecules (Figure 9b). There is competition between the oxygen molecules in the water and the oxygen molecules in the silica region as the simulation evolves; however, there is no longer a Ca-Si bond (Figure 9c). RDF was analyzed to clarify the Ca-O and Ca-Si interactions (Figure 10). The Ca-O peak is much larger and higher than the Ca-Si peak, unlike the first system studied. The Ca-Si peak occurs primarily after 3 Å, implying that the two atoms are not within bonding distance most of the time; therefore, Ca^2+^ would bond more to water molecules in the presence of water molecules. Ca^2+^ will still bind to the oxygen atoms in the silica region of PAM-Silica.

Summarily, Table 2 shows a list of all model systems calculated to explain how the calculations begin from DFT optimization and progress to AIMD. DFT optimization was initially done for all the oligomers followed by binding energy calculations. The workflow confirms how computational chemistry techniques such as DFT and AIMD can be employed in studying and validating a system. In this case, different oligomers were investigated and only those with PAM had good binding affinities with Ca^2+^. QTAIM analysis confirmed the binding interactions using the BCPs. Ca-PAM silica-II which had the highest binding energy with Ca^2+^, was further analyzed using AIMD to confirm further the way by which Ca^2+^ binds to the silicon atom in PAM-Silica-II. The findings of this work are in agreement with the recent work Sun et al. [58] though in their case the binding of Ca^2+^ with PAM was done with anionic PAM. However, their studies confirm the strong interaction of Ca with Oxygen atoms as observed in this work. 

## 4. Conclusions

The DFT studies indicate that Ca^2+^ does not bind readily to mixed oligomers of acryl acid and acrylamide; however, it has a good binding affinity with PAM and silica functionalized PAM, with the latter having a very high binding affinity. The highest binding affinity occurs in the silica region and not the –CONH functional groups. AIMD calculations corroborate the DFT studies since it was observed from the MD trajectory that Ca^2+^ binds to PAM-Silica by forming bonds with silicon. Ca^2+^ initially forms a bond with silicon in the presence of water molecules, but this does not last long; therefore, it subsequently bonds with the oxygen atoms present in the water molecule. PAM-Silica is a suitable calcium scale inhibitor since it has a high binding affinity with Ca^2+^; however, it will compete to capture the Ca^2+^ in the presence of water molecules. Theoretical studies (DFT and AIMD) have provided atomic insights on how PAM_Silica may be used as an efficient scale inhibitor; however, it may be further modified considering the competition from water molecules when used as a scale removal in an aqueous environment. Nevertheless, future studies may explore the use of the ReaxFF molecular dynamics technique which combines the accuracy of AIMD whilst enabling the analysis of larger number of atoms at longer time scales. Hence, building a more realistic model particularly with respect to the oligomer. However, the challenge is to ensure an existing Force Field that contains all the atoms to be studied exist in the near future this would be possible as ReaxFF continues to develop. 

## Figures and Tables

**Table 1:** The E_int_ energies derived from DFT and QTAIM calculations.

	Complex	ρ(r)	∇^2^ ρ(r)	H_b_	V(r)	G(r)	E_int_^a^	E_int_^b^
**BCP**	**Ca_PAM**							
68	Ca-H	0.015	0.062	0.002	−0.0109	0.013	3.42	3.55
70	Ca-O	0.040	0.207	0.003	−0.0458	0.049	14.42	13.19
80	Ca-N	0.043	0.174	0.001	−0.0417	0.043	13.13	11.52
	**Ca_PAM_hydro**							
80	Ca-O	0.050	0.270	0.003	−0.0598	0.063	18.84	17.05
77	Ca-O	0.050	0.279	0.004	−0.0611	0.065	19.24	17.68
	**Ca_PAM_SiO_2_-I**							
91	Ca-N	0.001	0.005	0.000	−0.0005	0.001	0.15	0.23
98	Ca-N	0.024	0.090	0.002	−0.0184	0.021	5.81	5.59
	**Ca_PAM_SiO_2_-II**							
77	Ca-N	0.013	0.005	0.000	−0.0005	0.0111	2.78	3.00
86	Ca-O	0.030	0.160	0.005	−0.0309	0.035	9.75	9.58
91	Ca-O	0.059	0.286	0.000	−0.0720	0.072	22.68	19.39

ρ(r) = Density of electrons; ∇^2^ ρ(r) = Laplacian of electron density, Hb = Energy density; V(r) = potential energy density (Hatrees); G(r) = Langragian Kinetic energy (Hatrees); Eint^a^ = [−V(r)/2] × 630 Kcal/mol [56]; Eint^b^ = 0.429 G(r) × 630 kcal/mol [57].

**Table 2:** Summary of model systems and calculation types.

Model System	Calculation Type	Program	Remark
AA-AA-AA-AM (70%-Acrylic Acid)	DFT Optimization	Gaussian 09	✓
AA-AA-AM-AM (50% each of acryl acid and acryl amide)	DFT Optimization	Gaussian 09	✓
AA-AM-AM-AM (70% acryl amide)	DFT Optimization	Gaussian 09	✓
PAM	DFT Optimization	Gaussian 09	✓
PAM_hydrolyzed	DFT Optimization	Gaussian 09	✓
PAM Silica	DFT Optimization	Gaussian 09	✓
Ca-AA-AA-AA-AM	Binding energy	Gaussian 09	O
Ca-AA-AA-AM-AM	Binding energy	Gaussian 09	O
Ca-AA-AM-AM-AM	Binding energy	Gaussian 09	O
Ca-PAM	Binding energy	Gaussian 09	✓
Ca-PAM_hydrolyzed	Binding energy	Gaussian 09	✓
Ca-PAM Silica-I	Binding energy	Gaussian 09	✓
Ca-PAM Silica-II	Binding energy	Gaussian 09	✓
Ca-PAM	QTAIM analysis	Multiwfn	✓
Ca-PAM_hydrolyzed	QTAIM analysis	Multiwfn	✓
Ca-PAM Silica-I	QTAIM analysis	Multiwfn	✓
Ca-PAM Silica-II	QTAIM analysis	Multiwfn	✓
Ca-PAM Silica-II	AIMD	VASP	✓

## Supplementary Materials

The following supporting information can be downloaded at: https://www.mdpi.com/article/10.3390/polym14122333/s1, Table S1. The DFT calculated energy values and binding energies of the studied complexes.
