# Supplementary material for: Theoretical Studies of a Silica Functionalized Acrylamide for Calcium Scale Inhibition
Source: Polymers (Basel). 2022 Jun 9;14(12):2333. doi: 10.3390/polym14122333 (PMC9230130; doi:10.3390/polym14122333)
Supplement: Supplementary file 1 [file polymers-14-02333-s001.zip › polymers-1690147-supplementary.pdf]

polymers

Theoretical studies of a Silica-Functionalized Polyacrylamide for Calcium Scale Inhibition

Abdulmujeeb T. Onawole [1], Ibnelwaleed A. Hussein [1,2,*], Mohammed A Saad [1,2], Nadhem Ismail [3], Ali Alshami [3,*] and Mustafa S. Nasser [1,2]

Table S1. The DFT calculated energy values and binding energies of the studied complexes.

Name	Energy (Hatrees)	Binding Energy (Hatrees)	Binding Energy(kJ/mol)
Ca	-677.466		
AA_AA_AA_AM	-1050.32		
AA_AA_AM_AM	-1030.43		
AA_AM_AM_AM	-1010.54		
Pure_PAM	-951.366		
PAM_hydro	-1029.12		
PAM_SiO2	-1580.54		
	**Complexes**		
Ca_AA_AA_AA_AM	-1726.47	1.308	820.463
Ca_AA_AA_AM_AM	-1707.05	0.846	530.815
Ca_AA_AM_AM_AM	-1687.73	0.282	177.096
Ca_Pure_PAM	-1629.00	**-0.169**	**-106.051**
Ca_PAM_hydro	-1707.05	**-0.461**	**-289.027**
Ca_PAM_SiO2	-2258.45	**-0.439**	**-275.301**
Ca_PAM_SiO2b	-2258.51	**-0.499**	**-313.431**
